# Editorial: Extracellular vesicles in cardiovascular inflammation and calcification

**DOI:** 10.3389/fcvm.2022.1077124

**Published:** 2022-11-08

**Authors:** Jona B. Krohn, Elena Aikawa, Masanori Aikawa, Joshua D. Hutcheson, Susmita Sahoo, Jason E. Fish

**Affiliations:** ^1^Department of Cardiology, Angiology and Pulmonology, University Hospital Heidelberg, Heidelberg, Germany; ^2^German Centre for Cardiovascular Research (DZHK), Partner Site Heidelberg/Mannheim, Heidelberg, Germany; ^3^Center for Interdisciplinary Cardiovascular Sciences, Division of Cardiovascular Medicine, Brigham and Women's Hospital, Harvard Medical School, Boston, MA, United States; ^4^Center for Excellence in Vascular Biology, Department of Medicine, Brigham and Women's Hospital, Harvard Medical School, Boston, MA, United States; ^5^Department of Biomedical Engineering, Florida International University, Miami, FL, United States; ^6^Biomolecular Sciences Institute, Florida International University, Miami, FL, United States; ^7^Cardiovascular Research Institute, Icahn School of Medicine at Mount Sinai, New York, NY, United States; ^8^Toronto General Hospital Research Institute, University Health Network, Toronto, ON, Canada; ^9^Department of Laboratory Medicine and Pathobiology, University of Toronto, Toronto, ON, Canada; ^10^Peter Munk Cardiac Centre, Toronto General Hospital, University Health Network, Toronto, ON, Canada

**Keywords:** extracellular vesicles (EV), vascular calcification, calcific aortic valve disease, coronary artery disease, atherosclerosis, inflammation

## Background to the research topic

In the age of evidence-based precision medicine, the exploration of novel therapeutic strategies for cardiovascular diseases such as atherosclerosis or calcific aortic valve disease (CAVD) has increasingly moved into the spotlight. Calcification of cardiovascular tissues is a harbinger of poor cardiovascular outcomes and has emerged as a therapeutic target (1). In the pathophysiology of calcific vascular and valvular disease, extracellular vesicles (EVs) play a central role in the initiation and progression of both fibrocalcific and inflammatory responses (2). These membrane-bound nano-sized entities released by various cell types carry selective proteins, small RNAs and calcium phosphate mineral, thus acting as messengers in cell-cell and cell-matrix communication. EVs have been shown to play key roles in all stages of cardiovascular disease, from initiation to the propagation of an inflammatory response leading to progressive calcification and its potentially fatal sequelae, including plaque rupture and heart failure (3, 4). Moreover, recent studies have introduced circulating EVs as novel biomarkers for the diagnosis and prognosis of various cardiovascular diseases.

The cumulative work summarized herein may help move us closer to individualized, target-specific therapies for CAVD and atherosclerosis.

## Emerging approaches to understand the role of EVs in cardiovascular inflammation and calcification

Limitations in single EV studies have precluded a thorough analysis of molecular cargo and function of EV subsets. Using a tailor-made EV microarray assay, Rogers et al. characterized the EVs produced by primary human smooth muscle cells and valvular interstitial cells in response to exposure to Lipopotein(a) [Lp(a)], a marker and potential mediator of cardiovascular calcification disease risk. They found that Lp(a) induced calcification of these cells and could modulate the abundance of secreted EVs through inflammatory signaling downstream of oxidized phospholipids. Single EV analysis showed that secretion of microvesicles containing CD29 but lacking tetraspanins were increased while secretion of exosomes from calcified cells was decreased. Functionally, the authors demonstrated that these EVs carried Annexins, which have previously been implicated in calcification (5) and specifically the induction of microcalcifications. This study links Lp(a) and calcification risk through its effect on EV production and function. Further studies are required to determine the subtypes of EVs that drive calcification.

A systems-wide approach integrating the metabolome and proteome could bridge a key knowledge gap to characterize the molecular mechanisms of CAVD. Fu et al. performed a metabolomic and proteomic comparison of valve samples from patients with CAVD and matched controls, which revealed numerous differentially enriched pathways. A metabolite-protein-pathway network was constructed, and hubs in the network were identified. While this study did not specifically investigate EV proteins, it is likely that the networks are impacted by EV-mediated cell–cell communication in the valve tissue microenvironment. Profiling the proteins and metabolites contained within tissue EVs in the setting of calcification would greatly add to our understanding of this network.

Alterations in the microenvironment may stimulate modulation of EV production and cargo loading, impacting EV function. Carter et al. sought to determine the impact of a pro-inflammatory cytokine milieu on the function of EVs released by endothelial cells from coronary artery and adipose tissue. They found that EV production increased and that subsets of EVs were altered under inflammatory conditions. These EVs have an angiostatic effect on coronary artery endothelial cells, reducing proliferation and compromising barrier integrity. While the mechanisms need further exploration, the authors identified microRNAs that are predicted to impact angiogenesis, proliferation and barrier properties. Their findings have implications for inefficient repair in a pro-inflammatory microenvironment. The concept that cellular state can dictate the functionality of secreted EVs was further discussed by Mas-Bargues et al.. They reviewed the emerging impact of EVs produced by senescent endothelial cells and smooth muscle cells on the process of vascular calcification. The authors concluded that inflammation and aging are key drivers of calcification, and that these processes are associated with senescence, a phenotype that is known to stimulate the production of pro-calcifying EVs. Further research is needed to fully understand how senescence and other cellular states impact cell-cell communication in the vessel or valve to initiate and promote calcification.

## Circulating EVs as independent predictors of calcific vascular disease and adverse cardiovascular outcome

EVs isolated from serum samples have previously been implicated in cardiac stress responses in the setting of various cardiovascular diseases (6). Moreover, specific subsets of circulating EVs were found to be quantitatively or qualitatively correlated with cardiovascular disease outcomes (7). Using flow cytometric analysis of EV isolates from patient blood samples, Siegel et al. identified patterns of differentially regulated EV subpopulations in a cohort of patients on veno-arterial extracorporeal membrane oxygenation (VA-ECMO) compared to patients with ST-elevation myocardial infarction (STEMI). Quantitative analysis revealed that Annexin V^+^ EV levels in serum predicted survival in patients on VA-ECMO, implicating a putative role of circulating EVs in the setting of severe organ dysfunction. In patients with STEMI, serum levels of cardiomyocyte-derived caveolin-3^+^ EVs correlated with severity of post-ischemic myocardial injury, providing further evidence that circulating EVs are useful biomarkers of myocardial ischemia.

In an effort to optimize diagnosis of coronary artery disease (CAD) using serum-derived EVs as biomarkers, Xiong et al. applied microarray analysis of messenger RNAs in PBMC-derived EVs from CAD patients compared to healthy controls. This study identified an upregulation of EV-bound mRNA for *S1RP5* and an inverse downregulation of *CARNS1* mRNA as independent predictors of angiographically diagnosed CAD. Both *S1RP5* and *CARNS1* have previously been linked to relevant downstream pathways in the pathogenesis of atherosclerosis, specifically inflammatory responses and lipid metabolism and angiogenesis (8, 9), respectively. In combination, expression levels of EV-bound *S1RP5* and *CARNS1* mRNA exhibited a slightly lower sensitivity and specificity for the diagnosis of CAD compared to coronary CT, while posing a more economic and procedurally safe diagnostic test.

## Summary and future perspectives

Our understanding of how cells communicate is continuing to evolve, and EVs are now thought to play a central role in dictating intercellular interactions. Knowledge gaps remain partly due to the technical challenges of interrogating EV biology in an intact vessel or valve. Future advances will require technological innovations, including visualization of EV trafficking in living intact tissues and single EV analytical approaches. The two translational studies included in our Research Topic add to a growing body of evidence highlighting EVs as a treasure trove of biomarker potential, and careful controls will be necessary to determine their diagnostic and prognostic capacity. Additional work is required to connect circulating markers to specific localized disease processes. Finally, an understanding of the mechanisms whereby EVs can modulate disease processes will guide the design of therapies that target or mimic these pathways. This is especially prescient for calcification, which does not have effective treatments.

## Author contributions

JK and JF conceived and wrote the editorial. EA, MA, JH, and SS wrote, edited, and approved of the editorial. All authors contributed to the article and approved of the submitted version.

## Conflict of interest

The authors declare that the research was conducted in the absence of any commercial or financial relationships that could be construed as a potential conflict of interest.

## Publisher's note

All claims expressed in this article are solely those of the authors and do not necessarily represent those of their affiliated organizations, or those of the publisher, the editors and the reviewers. Any product that may be evaluated in this article, or claim that may be made by its manufacturer, is not guaranteed or endorsed by the publisher.
